# Monthly Summary

**Published:** 1869-03

**Authors:** 


### MONTHLY SUMMARY.

*Chlorine Gas in Toughening and Refining of Gold.*—The methods now in use for effecting the above purposes are all more or less unsatisfactory. Mr. F. B. Miller, F.C.S., Assayer in the Sydney branch of the Royal Mint, has therefore devised a process which appears to satisfy all the requirements of the case in a single operation.

A French clay crucible is saturated with borax by immersing it in a hot saturated solution, and drying. The gold is then melted in this crucible with a little borax, and a stream of chlorine gas is allowed to pass through it by means of a clay tube (a tobacco pipe stem was found suitable.) The chlorine generator is fitted with a safety tube 7 feet long, and is connected with a clay tube by a caoutchouc tube. In a few hours the whole of the silver is converted into chloride, which floats on the gold. The borax prevents the absorption of the chloride by the crucible, and also its volatilization, except in very minute quantities. As soon as the gold has become solid, the still liquid chloride of silver is poured off, and the gold is now found to have a fineness of say 993 parts in 1,000. The apparent loss of gold is very little greater than is found in ordinary gold melting—being 2·9 parts in 10,000—whereas in the ordinary process it is 2. A small sample of the gold is removed from time to time during the operation by means of a piece of tobacco-pipe used as a pipette. This is rapidly assayed approximately, and thus the progress of the operation is judged of.

The fusil chloride of silves obtained as a slag after the operation, is reduced by placing it between two plates of wrought-iron in a bath of dilute sulphuric acid. The spongy silver so obtained contains gold, which may be separated by nitric acid. The nitrate of silver can of course be precipitated as chloride, and subsequently reduced. The gold appears to be present in the chloride of silver in the form of a double chloride, and Mr. Miller has succeeded in separating it directly from this combination by precipitation by metallic silver.—*Druggists' Circular.*

